# Phosphoinositide 3-Kinase Promotes Oxidative Burst, Stomatal Closure and Plant Immunity in Bacterial Invasion

**DOI:** 10.3389/fpls.2019.01740

**Published:** 2020-01-24

**Authors:** Huiying Zhang, Xin Liu, Xiyong Zhang, Ningning Qin, Kaifang Xu, Weihua Yin, Yueqin Zheng, Yuanyuan Song, Rensen Zeng, Jian Liu

**Affiliations:** ^1^Fujian Provincial Key Laboratory of Plant Functional Biology, College of Life Sciences, Fujian Agriculture and Forestry University, Fuzhou, China; ^2^Key Laboratory of Ministry of Education for Genetics, Breeding and Multiple Utilization of Crops, College of Agriculture, Fujian Agriculture and Forestry University, Fuzhou, China

**Keywords:** phosphoinositide 3-kinase, stomatal immunity, reactive oxygen species, phytohormone, *PR* gene1

## Abstract

Phosphoinositide 3-kinase (PI3K) plays a vital role in plant response to abiotic stress. However, the role of PI3K in plant immunity is largely unknown. This study showed that PI3K enhanced *Arabidopsis* resistance to *Pseudomonas syringae* pv *tomato* DC3000 (*Pst* DC3000) and *Pst* DC3000 (*avrRpt2*). Overexpression of *AtVPS34* promoted stomatal closure while PI3K inhibitors blocked that after spray inoculation. Additionally, gene expression of *AtVPS34* was increased upon infection by *Pst* DC3000 (*avrRpt2*), and SA upregulated *AtVPS34* gene expression in this process. Furthermore, overexpression of *AtVPS34* enhanced *PR* gene expression after syringe infiltration with *Pst* DC3000 (*avrRpt2*), while PI3K inhibitors inhibited that. The production of hydrogen peroxide and the expression of gene encoding antioxidant enzyme were both enhanced in *AtVPS34* overexpressing lines after spray inoculation or syringe infiltration with *Pst* DC3000 (*avrRpt2*). Collectively, these results unraveled a novel and broad role of PI3K in plant immunity which promoted stomatal closure and *PR* gene expression possibly *via* regulating ROS production.

## Introduction

Plant defense involves two overlapping tiers of responses, PAMP-triggered immunity (PTI) and effector-triggered immunity (ETI) (Jones and Dangl, 2006). PTI involves distinct well-characterized physiological mechanisms, such as stomata closure to limit pathogen entry, reactive oxygen species (ROS) production, the biosynthesis of antimicrobial metabolites and proteins such as pathogenesis-related (PR) proteins, defense hormones such as salicylic acid (SA), jasmonic acid (JA). In contrast to PTI, ETI induces stronger and long-lasting responses, which is frequently accompanied by programmed cell death, a process known as the hypersensitive response (HR), leading to pathogen resistance. Autophagy operates negative feedback loop modulating SA signaling to suppress the process of HR (Yoshimoto et al., 2009). Phosphoinositide 3-kinase (PI3K) as a key regulator of lipid signal has been reported to be involved in autophagy. PI3K is associated with BECN1/ATG6 to construct the PI3K type III complex, contributing to activation of autophagy (Abeliovich and Klionsky, 2001). In mammalian cells, there are three types of PI3K with distinct substrate specificities. In cells of the mammalian immune system, PI3K is activated by receptors for antigen, cytokines, costimulatory molecules, and so on. PI3K signaling regulates immune cell proliferation, survival, differentiation, chemotaxis, phagocytosis, degranulation, and respiratory burst (Fruman and Cantley, 2002). However, type III PI3K (VPS34), the only type of PI3K identified in plants, phosphorylates the D-3 position of phosphoinositides to generate phosphatidylinositol-3-phosphate (PI3P) (Bunney et al., 2000). In *Arabidopsis*, PI3K is encoded by a single-copy gene, *AtVPS34* (Lee et al., 2008). Given its evolutionarily conserved family of signal transducing enzymes, it is worth revealing the role of PI3K/VPS34 in plant immunity.

Actually, phosphoinositide signaling plays a vital role in plant immunity. The abundant types and metabolizing enzymes of phosphoinositide allowing rapid and reversible interconversion between them provide a highly dynamic and powerful system to coordinate membrane reorganization, vesicle trafficking and cytoskeleton rearrangements as well as signaling pathways determining cell fate (Payrastre et al., 2012). Meanwhile, pathogens have evolved many different strategies to subvert the phosphoinositide metabolism and express phosphoinositide binding effectors (Payrastre et al., 2012). In the process of oomycetes infection, effector *Avh5* could interacts with PI3P, which promotes host cell entry (Kale et al., 2010). After internalization, PI3P assists bacterial toxins to escape *via* several mechanisms including retrograde translocation from the ER and transit of partially unfolded proteins directly across membranes (Bhattacharjee et al., 2012). However, PI3K also seems to play a positive role in immune response. PI3K/VPS34 functions not only to limit the spread of TMV-induced HR PCD, but also to inhibit virus replication and/or movement (Liu et al., 2005). In addition, PI3P activates the p40^phox^ subunit of NADPH oxidase that forms part of the human innate immune response (Ellson et al., 2006). Therefore, the role of PI3K in immune response is complex. Despite many reports, the role of PI3K is still not entirely clear besides its role in HR. For example, PI3K/AtVPS34 functions in stomatal closure in plant stress response (Jung et al., 2002; Park et al., 2003; Choi et al., 2008; Liu et al., 2016), indicating a possible role of PI3K/AtVPS34 in stomatal immunity.

In this study, two types of bacteria, *Pseudomonas syringae* pv *Tomato* DC3000 (*Pst* DC3000) and avirulent *Pst* DC3000 (*avrRpt2*), were used. To examine the novel role of PI3K in plant immunity, *AtVPS34* overexpressing *Arabidopsis* and two PI3K inhibitors, LY294002 (LY) and wortmannin (WM), were used as previously described (Liu et al., 2016). LY, which is derived from the flavonoid quercetin, competes with ATP and binds to Lys residues in the ATP-binding pocket of PI3Ks (Walker et al., 2000). WM, a fungal metabolite, dose-dependently targets PI3K and PI4K (Takáč et al., 2012). Given the key role of PI3K in stomatal movement, stomatal aperture was determined after spray inoculation. The effect of PI3K on plant immunity after syringe infiltration was also examined by phenotype analysis, *PR* gene expression and ROS signaling. In conclusion, we revealed a new role of PI3K during bacterial infection in *Arabidopsis*.

## Materials and Methods

### Plant Material

Seedling of wild-type (ecotype Columbia) *Arabidopsis* (*Arabidopsis thaliana*), transgenic *PR1pro::GUS Arabidopsis* (Sun et al., 2012), 35Spro::YFP *Arabidopsis* and 35Spro::*AtVP34*-YFP *Arabidopsis* were sterilized and grown in soil as described previously (Liu et al., 2016). *AtVPS34* was cloned into the pHB-YFP vector containing CaMV35S promoter to generate 35Spro::*AtVPS34*-YFP plasmid (Liu et al., 2016). The determination of transgenic *Arabidopsis* was shown in **Figure S1**.

### Chemicals

Commercial chemicals were used at the following concentrations: 30 μM LY (Beyotime), 10 μM WM (Beyotime), 10 μM abscisic acid (ABA, Beyotime), 1 mM salicylic acid (SA, Sigma), 1 mM methyl jasmonate (MeJA, Sigma), 20 μM brassinolide (BL, Sigma), 100 μM indol-yl-3-acetic acid (IAA, Sigma), and 10 μM gibberellin (GA, Sigma). The treatment of phytohormone was performed according to the previous study (Yi et al., 2014; Yuan et al., 2017).

### Pathogen Growth and Inoculation

Bacterial infections were performed with three-week-old *Arabidopsis*. *Pst* DC3000 (*avrRpt2*) and *Pst* DC3000 was cultured at 30°C in LB medium supplemented with appropriate antibiotics (Melotto et al., 2006; Hung et al., 2014). Overnight log-phase cultures were cultured by centrifugation, washed with 10 mM MgCl_2_, and then diluted to a final optical density at 600 nm (OD600) of 0.01. The bacterial suspensions were infiltrated from the abaxial side into a leaf using a 1 ml syringe without needle.

For stomatal immunity assay, 3-week-old *Arabidopsis* were uniformly spray-inoculated with *Pst* DC3000 suspension (OD600 = 0.05) in 10 mM MgCl_2_ with 0.02% Silwet L-77 (Su et al., 2017).

### Bacterial Growth Assay

The harvested leaves were surface sterilized (30 s in 70% ethanol, then 30 s in sterile distilled water) for spray inoculation (Zipfel et al., 2004). Three leaf discs (5 mm diameter) containing the sites syringe-infiltrated or spray-inoculated with bacteria were excised from each leaf and collected in three groups, each of which contained six discs each from a different leaf (Alvarez et al., 1998). Leaf discs were ground in 10 mM MgCl_2_, then thoroughly vortex-mixed and diluted 1:10 serially. Samples were finally plated on King's B medium with appropriate antibiotics. Colonies were counted after incubation at 30°C.

### Assessment of Response of Stomata to Treatments

To assure that most stomata were open before beginning experiments, we kept plants under light (100 μE/m^2^/s) for at least 3 h. Full expanded young leaves were immersed in water or bacterial suspension (10^8^ cfu/ml in water). 1 and 3 hours after spray inoculation, epidermis of three leaves was peeled off and immediately observed by a microscope. The width and length of the stomatal aperture were measured using the software ImageJ. All stomatal aperture results reported here were from blind experiments in which genotypes and treatments were unknown to the experimenters who measured stomatal responses until the completion of experiments (Melotto et al., 2006).

### RNA Extraction and RT-PCR Analyses

Total RNA was extracted using RNAiso Plus kit (Takara, cat. no. 9108) according to the manufacturer's specifications. cDNA synthesis was carried out using GoScript™ reverse transcription system (Promega, cat. no. A5000).

The transcript levels of *AtVPS34*, *AtPR1*, *AtPR5, AtCAT1, AtCAT2, AtAPX1, AtCSD1, and AtMSD1* genes were analyzed by quantitative RT-PCR. The gene-specific primers were shown in **Table 1**. *AtActin2* gene was amplified as a quantitative control.

**Table 1:** List of primers used in this study.

Name	Target gene	Sequences (5'→3')
*AtVPS34*-F	*AtVPS34*	GGTGTTAGCAACTGGACATGACG
*AtVPS34*-R		CAAGTGGCTGTTATCCCGAAAG
*AtPR1*-F	*AtPR1*	GGAGCTACGCAGAACAACTAAGA
*AtPR1*-R		CCCACGAGG ATCATAGTTGCAACTGA
*AtPR5*-F	*AtPR5*	CGGTACAAGTGAAGGTGCTCGTT
*AtPR5*-R		GCCTCGTAGATGGTTACAATGTCA
*AtCAT1*-F	*AtCAT1*	AAGTGCTTCATCGGGAAGGA
*AtCAT1*-R		CTTCAACAAAACGCTTCACGA
*AtCAT2*-F	*AtCAT2*	TCCGCCTGCTGTCTGTTCTG
*AtCAT2*-R		TGGGTCGGATAGGGCATCAA
*AtAPX1*-F	*AtAPX1*	ACTCTGGGACGATGCCACAAG
*AtAPX1*-R		TCTCGACCAAAGGACGGAAAA
*AtCSD1*-F	*AtCSD1*	TCCATGCAGACCCTGATGAC
*AtCSD1*-R		CCTGGAGACCAATGATGCC
*AtMSD1*-F	*AtMSD1*	ATGTTTGGGAGCACGCCTAC
*AtMSD1*-R		AACCTCGCTTGCATATTTCCA
*AtActin2* -F	*AtActin2*	TCTTCTCATCATCTATATCACGATC
*AtActin2* -R		TAAAAAAACGAGGTCAATGCG

### Histochemical GUS Staining

Approximately 3-week-old transgenic *PR1pro::GUS Arabidopsis* was syringe-infiltrated with *Pst* DC3000 (*avrRpt2*), 0, 12, and 24 h after infiltration, histochemical detection of GUS enzyme activity was performed as described by β-Galactosidase Reporter Gene Staining Kit (Solarbio, cat. no. G3060).

### Measurements of H_2_O_2_ Content

1 and 3 h after infection, syringe-infiltrated and spray-inoculated leaves were collected, then the content of H_2_O_2_ produced in leaf tissues was measured by Hydrogen Peroxide Assay Kit (Beyotime, cat. no. S0038).

### YFP Fluorescence Analysis

All microscopic observations were performed using a confocal laser-scanning microscope (Leica SP8). 35Spro::*AtVP34*-YFP-1 *Arabidopsis* was pretreated with 1 mM SA or not, then syringe-infiltrated with *Pst* DC3000 (*avrRpt2*). YFP fluorescence was examined one day after infiltration (YFP: excitation 514 nm, emission 525–550 nm).

### Western Blot and Coomassie Staining of Proteins

35Spro::*AtVP34*-YFP-1 *Arabidopsis* was pretreated with 1 mM SA or not, then syringe-infiltrated with *Pst* DC3000 (*avrRpt2*). One day after infiltration with *Pst* DC3000 (*avrRpt2*), the infected leaves were collected. The total protein were extracted from leaf samples. Proteins extracts were separated on a 12% (w/v) SDS-PAGE and transferred onto PVDF membranes, then subjected to immunodetection with GFP polyclonal antibody (Beyotime, AG279). The antigen-antibody complex was visualized with anti-rabbit secondary antibody and enhanced chemiluminescence. Coomassie staining of the large subunit of Rubisco was used as a loading control. All the experiments took three independent repetitions.

### Statistical Analysis

All assays were performed independently for a minimum of three biological replications. Data are represented as mean ± SD. Statistical analysis was performed with the Student’s paired t test.

## Results

### PI3K Functioned in Plant Immunity During *Pst* DC3000 (*AvrRpt2*) and *Pst* DC3000 Infection

**Figure 1:**
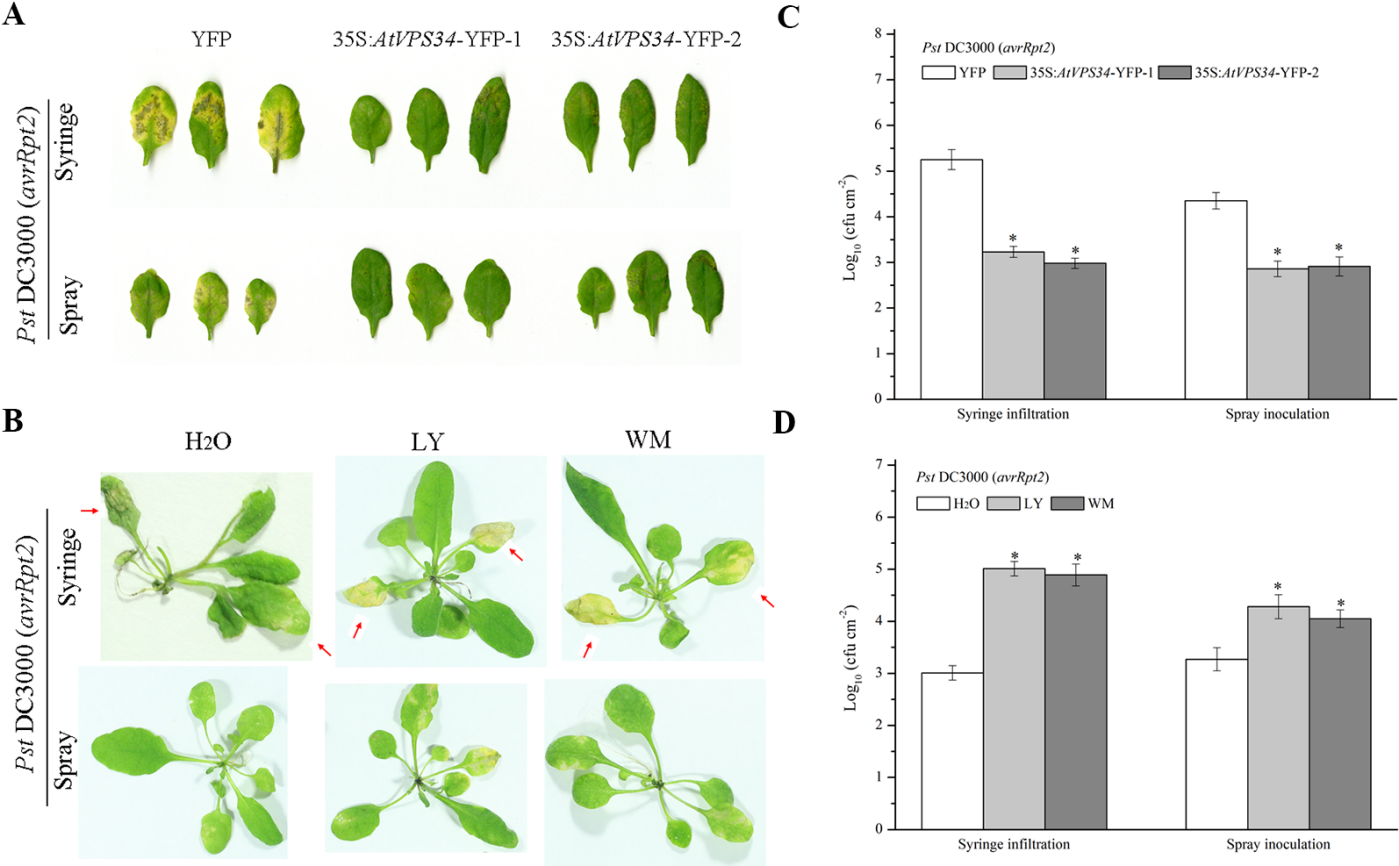
PI3K functioned in plant immunity against *Pst* DC3000 (*avrRpt2*). **(A)** 35Spro::*AtVP34*-YFP *Arabidopsis* and 35Spro::YFP *Arabidopsis* were spray-inoculated or syringe-infiltrated with *Pst* DC3000 (*avrRpt2*) for 3 days, Lesion phenotypes in *Arabidopsis* leaves was taken. Pictures represent typical examples. And the corresponding bacterial growth quantification of spray-inoculated or syringe-infiltrated leaves was shown in **(C)**. **(B)** WT *Arabidopsis* was pretreated either with 30 μM LY or 10 μM WM or not for 1 day, then syringe-infiltrated with *Pst* DC3000 (*avrRpt2*) for 1 day or spray-inoculated with *Pst* DC3000 (*avrRpt2*) for 2 days. Lesion phenotypes in *Arabidopsis* leaves was taken. The red arrow indicated the leaves infiltrated. Pictures represent typical examples. The corresponding bacterial growth quantification of spray-inoculated or syringe-infiltrated leaves was shown in **(D)**. Each bar is the mean ± SD of three biological replications. Asterisks (*) indicates significant difference by t test from 35Spro::YFP *Arabidopsis* or no PI3K inhibitors treatment (P < 0.05; Student's t test). PI3K, phosphoinositide 3-kinase; LY, LY294002; WM, wortmannin.

To assess the role of *PI3K* in plant immunity, a previous constructed *Arabidopsis* overexpressing *AtVPS34* was used (Liu et al., 2016). 35Spro::*AtVP34*-YFP lines showed more resistant compared with 35Spro::YFP *Arabidopsis* when syringe-infiltrated or spray-inoculated with *Pst* DC3000 (*avrRpt2*) (**Figures 1A, C**) and *Pst* DC3000 (**Figures S2A, C**). To further examine the role of PI3K in *Arabidopsis* resistance against bacteria invasion, two PI3K inhibitors LY and wortmannin (WM) was used. The specificity of LY and WM in inhibiting plant PI3K was shown in previous studies (Takáč et al., 2012; Leprince et al., 2015). *Arabidopsis* was pretreated with 30 μM LY or 10 μM WM for 24 h, then syringe-infiltrated or spray-inoculated with *Pst* DC3000 (*avrRpt2*) and *Pst* DC3000. More susceptible phenotype was observed on leaves after spraying with PI3K inhibitors compared with that in control (**Figures 1B, D** and **Figures S2B**, **D**). These results suggested that PI3K was involved in plant immunity against *Pst* DC3000 and *Pst* DC3000 (*avrRpt2*) invasion.

### PI3K Functioned in Stomatal Immunity During *Pst* DC3000 (*AvrRpt2*) and *Pst* DC3000 Infection

**Figure 2:**
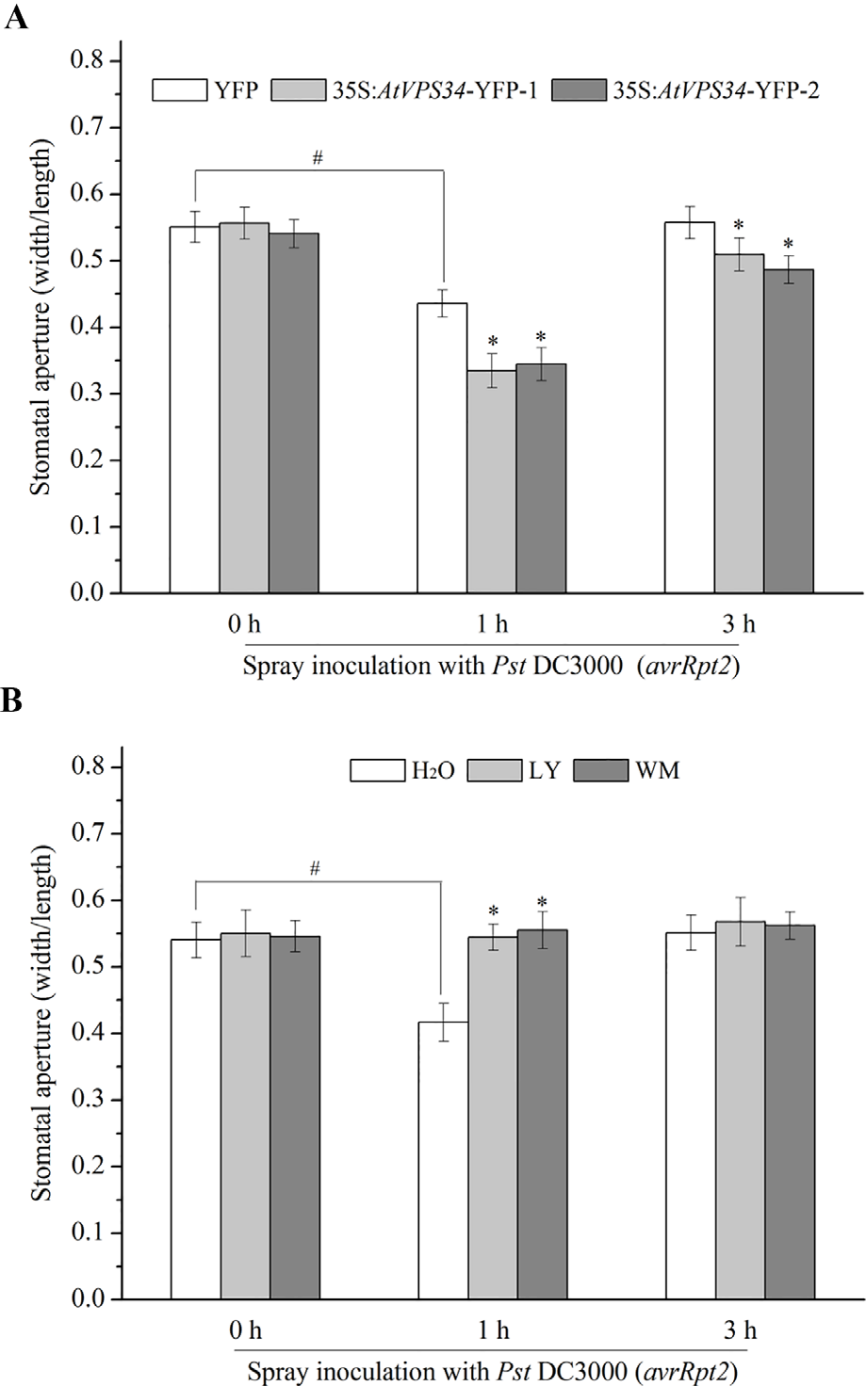
PI3K functioned in stomatal immunity against *Pst* DC3000 (*avrRpt2*). **(A)** 35Spro::*AtVP34*-YFP *Arabidopsis* and 35Spro::YFP *Arabidopsis* were taken in light for at least 3 h, **(B)** WT *Arabidopsis* was pretreated either with 30 μM LY or 10 μM WM or not for 24 h, then transport the plant under light for at least 3 h. The full expanded young leaves were immersed in water or *Pst* DC3000 (*avrRpt2*) suspension (10^8^ cfu/ml in water). 1 and 3 h after spray inoculation, epidermis of three leaves was peeled off and immediately observed under a microscope. The stomatal aperture was represented as the ratio of width to length. Each bar is the mean ± SD of three biological replications (n > 30). Asterisks (*) indicate statistically significant differences from control in the indicated times (P < 0.05; Student's t test). Hash marks (#) indicate statistically significant differences between indicated samples (P < 0.05; Student's t test). PI3K, phosphoinositide 3-kinase; LY, LY294002; WM, wortmannin.

Stomata are the first line of defense that prevents bacterial infection (Melotto et al., 2017). In previous study, PI3K was proved to participate in regulation of stomatal movement (Liu et al., 2005; Kale et al., 2010; Bhattacharjee et al., 2012). For clarify the possible role of PI3K in stomatal immunity, 35Spro::YFP *Arabidopsis* and 35Spro::*AtVP34*-YFP *Arabidopsis* were exposed to light for at least 3 h to enlarge the stomatal aperture, and then sprayed *Arabidopsis* leaves with *Pst* DC3000 (*avrRpt2*) or *Pst* DC30000. Stomatal aperture was determined 1 and 3 h after spray inoculation. It was shown that *Pst* DC3000 (*avrRpt2*) and *Pst* DC3000 induced stomatal closure at 1 hour and recovered at 3 h after spray inoculation. Additionally, no significant difference of stomatal aperture was found between 35Spro::*AtVP34*-YFP lines and 35Spro::YFP *Arabidopsis* with water treatment (**Figure 2A**). However, stomatal closure was enhanced in 35Spro::YFP *Arabidopsis* lines compared with that in 35Spro::YFP *Arabidopsis*, when spray-inoculated with *Pst* DC3000 (*avrRpt2*) or *Pst* DC3000 (**Figure 2A** and **Figure S3A**). And PI3K inhibitors blocked stomatal closure in stomatal immunity (**Figure 2B** and **Figure S3B**). These results indicated that PI3K was involved in stomatal immunity against *Pst* DC3000 (*avrRpt2*) and *Pst* DC3000 infection.

### Effect of Exogenous Phytohormone Supply on *AtVPS34* Expression When Syringe-Infiltrated With *Pst* DC3000 (*AvrRpt2*)

**Figure 3:**
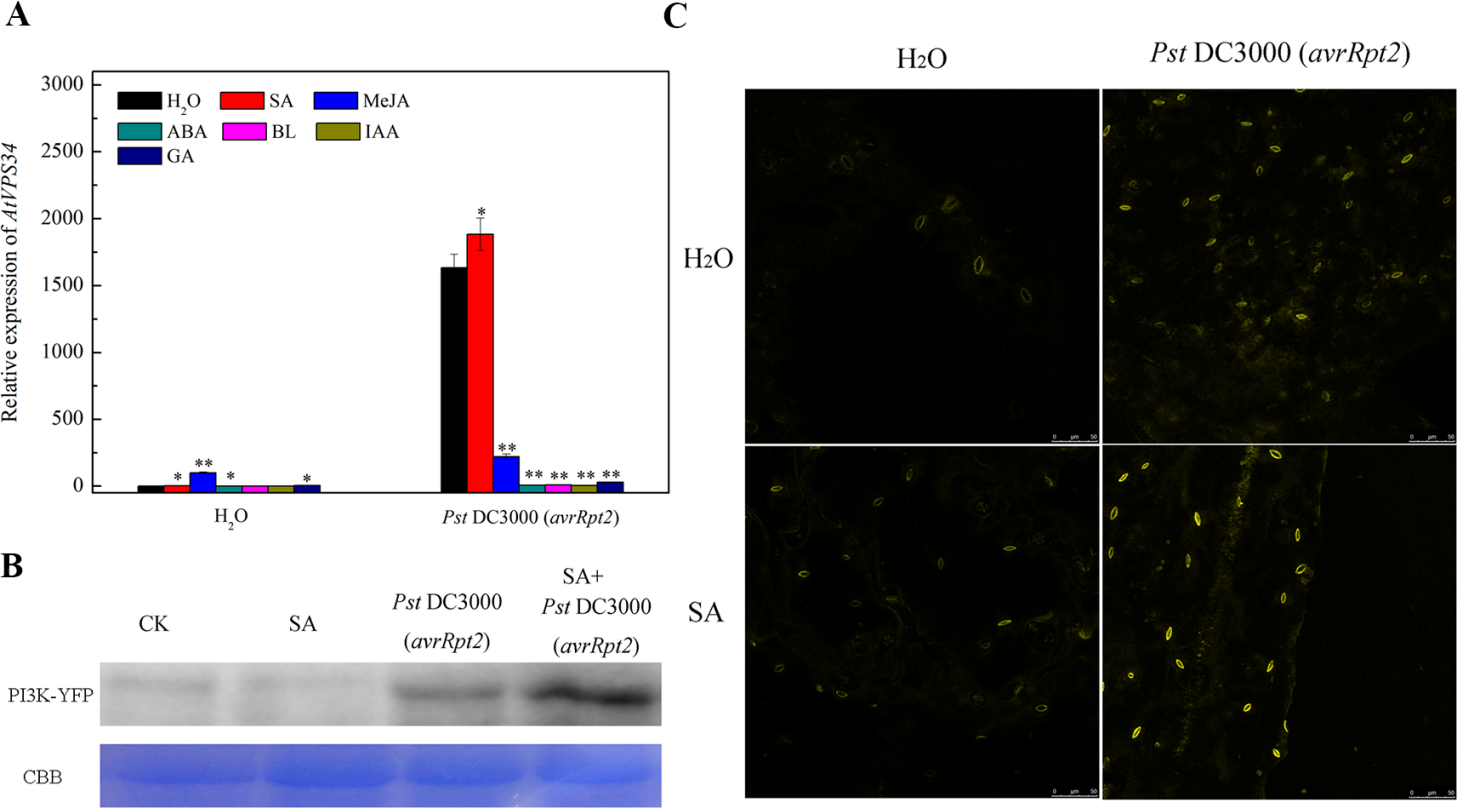
The effect of exogenous phytohormone supply on *AtVPS34* gene expression against *Pst* DC3000 (*avrRpt2*). **(A)** three-week-old *Arabidopsis* leaves were pretreated either with water (H_2_O) or 10 μM abscisic acid (ABA) or 1 mM salicylic acid (SA) or 1 mM methyl jasmonate (MeJA) or 20 μM brassinolide (BL) or 100 μM indol-yl-3-acetic acid (IAA) or 10 μM gibberellin (GA) for 1 day, then syringe-infiltrated with *Pst* DC3000 (*avrRpt2*). 12 h after infiltration, RNA was extracted from leaves, *AtVPS34* gene expression was examined by qPCR. *AtActin2* was used as an internal control. Each bar is the mean ± SD of three biological replications. Asterisks (*) indicate a significant difference from the treatment with water at *P < 0.05 or **P < 0.01 by *t*-test. **(B)** and **(C)** 35Spro::*AtVP34*-YFP-1 *Arabidopsis* was pretreated with 1 mM SA or not, then syringe-infiltrated with *Pst* DC3000 (*avrRpt2*). Western blots showing PI3K levels 1 day after infiltration **(B)**. Coomassie staining of the large subunit of Rubisco was used as a loading control. CBB, Coomassie brilliant blue staining. **(C)** YFP fluorescence was also examined one day after infiltration. Results shown are representative. PI3K, phosphoinositide 3-kinase.

Phytohormones play a vital role in plant resistance against bacterial invasion. Previous studies revealed that PI3K functioned as a common platform for multi-hormone signaling to trigger intracellular response (Hirsch et al., 2007). To investigate the link PI3K and phytohormone in plant immunity, *Arabidopsis* leaves were pretreated either with water, 10 μM ABA, 1 mM SA, 1 mM MeJA, 20 μM BL, 100 μM IAA or 10 μM GA for 24 h, then syringe-infiltrated with *Pst* DC3000 (*avrRpt2*). Gene expression of *AtVPS34* was strongly induced after syringe inoculation with avirulent *Pst* DC3000 (*avrRpt2*) for 12 h compared with that in water treatment. Although the supply of exogenous phytohormone such as SA, MeJA, ABA, and GA promoted *AtVPS34* gene expression when syringe-infiltrated with water, only SA significantly increased *AtVPS34* gene expression while other phytohormones suppressed it after syringe inoculation with avirulent *Pst* DC3000 (*avrRpt2*) compared with that in no phytohormone treatment (**Figure 3A**).

To further investigate the role of SA on PI3K signaling activation in response to bacterial infection, 35Spro::*AtVP34*-YFP-1 *Arabidopsis* was pretreated with 1 mM SA or not, then syringe-infiltrated with *Pst* DC3000 (*avrRpt2*). The expression of PI3K was examined by western blot (**Figure 3B**) and fluorescent analysis (**Figure 3C**) 1 day after infiltration. The expression of PI3K was enhanced after *Pst* DC3000 (*avrRpt2*) infiltration, and exogenous SA further increased PI3K expression in bacterial invasion. These results indicated that SA induce PI3K signaling in plant resistance against *Pst* DC3000 (*avrRpt2*) invasion.

### PI3K Enhanced Pathogenesis Related Genes Expression in Plant Resistance Against *Pst* DC3000 (*AvrRpt2*) Invasion

**Figure 4:**
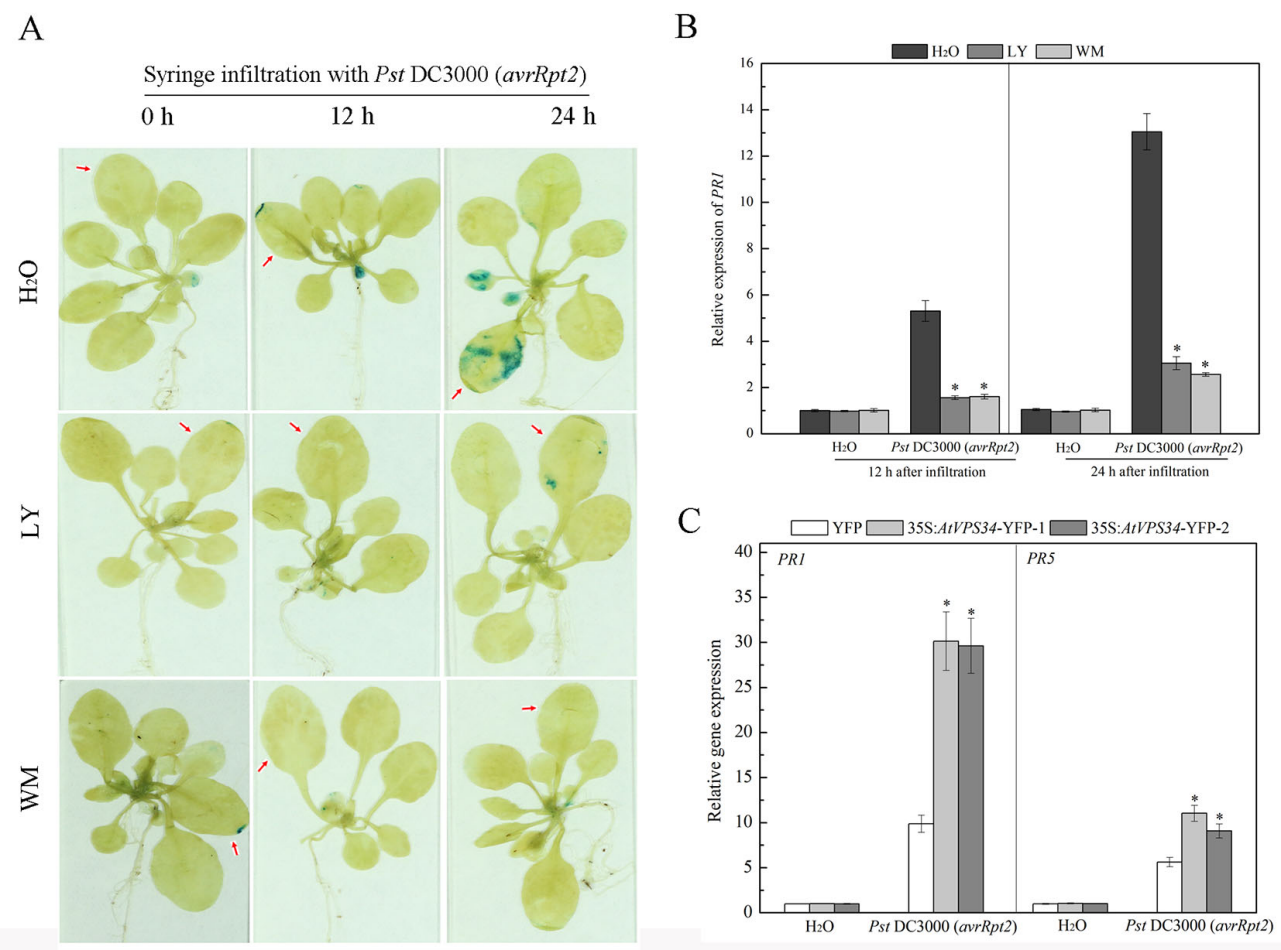
PI3K enhanced pathogen-related genes expression in resistance against *Pst* DC3000 (*avrRpt2*) invasion. **(A)** Approximately 3-week-old transgenic *PR1pro::GUS* Arabidopsis was syringe-infiltrated with *Pst* DC3000 (*avrRpt2*), 12 and 24 h after inoculation, histochemical detection of GUS enzyme activity was performed. The red arrow indicated the leaves infiltrated. Results shown are representative. **(B)** WT *Arabidopsis* was pretreated either with 30 μM LY or 10 μM WM or not for 24 h, then syringe-infiltrated with *Pst* DC3000 (*avrRpt2*) for 12 h or 24 h, *AtPR1* gene expression was examined by qPCR. Each bar is the mean ± SD of three biological replications. Asterisks (*) indicate statistically significant differences from the treatment with water (P < 0.05; Student's t test). **(C)** 35Spro::*AtVP34*-YFP *Arabidopsis* and 35Spro::YFP *Arabidopsis* were syringe-infiltrated with *Pst* DC3000 (*avrRpt2*) for 24 h, gene expression of *AtPR1* and *AtPR5* was determined by qPCR. Each bar is the mean ± SD of three biological replications. Asterisks (*) indicate statistically significant differences from 35Spro::YFP *Arabidopsis* (P < 0.05; Student's t test). PI3K, phosphoinositide 3-kinase; LY, LY294002; WM, wortmannin.

Pathogenesis related (PR) *PR1 and PR5* genes are late marker genes for *Arabidopsis* defense response (van Loon et al., 2006). Transgenic *PR1pro::GUS Arabidopsis* plants were used to examine *PR1* gene expression. In the group without PI3K inhibitors pretreatment, syringe infiltration with *Pst* DC3000 (*avrRpt2*) induced GUS signal at 12 h after infiltration and GUS signal was enhanced at 24 h after infiltration. In contrast, PI3K inhibitors inhibited *PR1* gene expression after syringe infiltration with *Pst* DC3000 (*avrRpt2*) in the first 24 h (**Figure 4A**). Moreover, similar results were found for PR1 gene expression by RT-qPCR analysis (**Figure 4B**).

*PR* genes expression was also examined in *AtVPS34* overexpressing *Arabidopsis* under bacterial infection. As shown in **Figure 4C**, overexpression of *AtVPS34* enhanced *PR1* and *PR5* gene expression compared with that in 35Spro::YFP *Arabidopsis* after syringe infiltration with *Pst* DC3000 (*avrRpt2*). These results indicated that PI3K had a positive role in transcription response of *PR* genes in plant resistance against *Pst* DC3000 (*avrRpt2*) invasion.

### PI3K Induced ROS Signaling in Plant Resistance Against *Pst* DC3000 (*AvrRpt2*) Invasion

**Figure 5:**
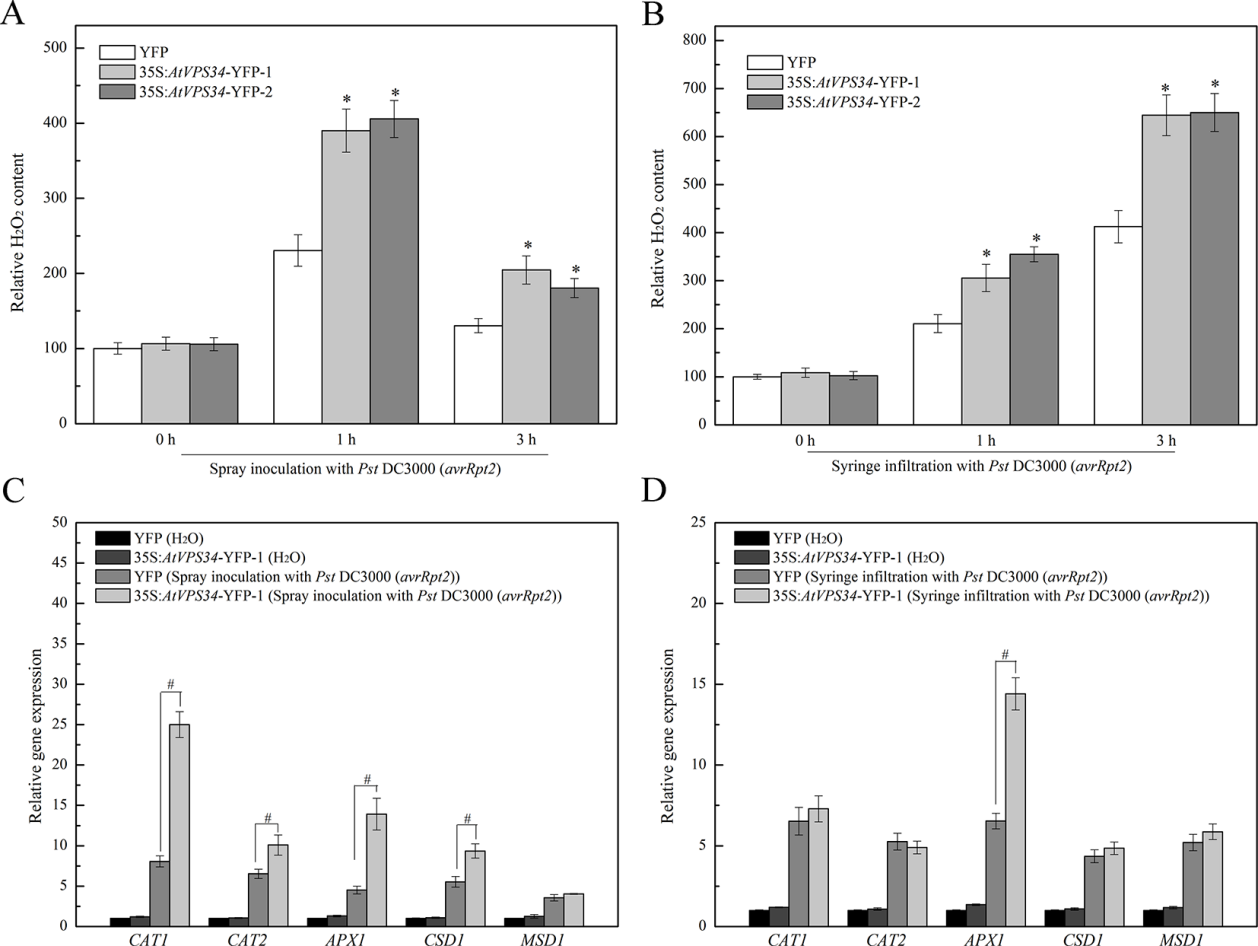
PI3K induced ROS signaling in *Arabidopsis* resistance against *Pst* DC3000 (*avrRpt2*) invasion. 1 and 3 h after invasion, the spray-inoculated **(A)** and syringe-infiltrated **(B)** leaves of 35Spro::*AtVP34*-YFP *Arabidopsis* and 35Spro::YFP *Arabidopsis* were collected, the H_2_O_2_ content was assayed using a colorimetric hydrogen peroxide assay kit from Beyotime. **(C**, **D)** 35Spro::*AtVP34*-YFP *Arabidopsis* and 35Spro::YFP *Arabidopsis* were spray-inoculated or syringe-infiltrated with *Pst* DC3000 (*avrRpt2*) for 24 h, then *CAT1*, *CAT2*, *APX1*, *CSD1*, and *MSD1* genes expression were determined by qPCR. Each bar is the mean ± SD of three biological replications. Hash marks (#) indicate statistically significant differences between indicated samples (P < 0.05; Student's t test). PI3K, phosphoinositide 3-kinase.

Previous studies have revealed that ROS plays a vital role in plant immunity (Lambeth, 2004) and PI3K regulates ROS production in various physiological events and stress responses (Lee et al., 2010). Therefore, 1 and 3 h after bacteria invasion, syringe-infiltrated and spray-inoculated leaves were collected. As shown in **Figure 5**, the content of hydrogen peroxide was significantly increased at 1 h after spray-inoculation and declined at 3 h after spray-inoculation in 35Spro::YFP *Arabidopsis*, while syringe infiltration with *Pst* DC3000 (*avrRpt2*) induced a progressive increase of hydrogen peroxide. However, overexpression of *AtVPS34* further upregulated the content of hydrogen peroxide compared with that in 35Spro::YFP *Arabidopsis* after bacterial invasion.

Additionally, to further reveal the key role of PI3K in ROS signaling, the expression of genes encoding antioxidant enzymes was also examined. Most of genes encoding antioxidant enzymes were upregulated after spray inoculation with *Pst* DC3000 (*avrRpt2*) (**Figure 5C**), and gene expression of *APX1* was increased after syringe infiltration with *Pst* DC3000 (*avrRpt2*) (**Figure 5D**). It seems that PI3K was involved in plant immunity *via* promoting ROS production.

## Discussion

In this study, we investigated the role of PI3K in plant immunity against *Pst* DC3000 (*avrRpt2*). Overexpression of *AtVPS34* enhanced plant resistance against *Pst* DC3000 and *Pst* DC3000 (*avrRpt2*) invasion. Further analysis showed that stomatal immunity was enhanced by the overexpression of *AtVPS34*. In addition, exogenous SA treatment enhanced *AtVPS34* expression and *PR* gene expression was significantly induced by the overexpression of *AtVPS34* after syringe infiltration with *Pst* DC3000 (*avrRpt2*). Moreover, the content of hydrogen peroxide and the expression of genes encoding antioxidant enzyme were increased when inoculated with *Pst* DC3000 (*avrRpt2*) regardless of syringe or spray. These results indicated a novel role of PI3K in plant immunity.

Plant effective immune responses are activated through various phytohormones signaling such as jasmonic acid, ethylene, Abscisic acid, auxin, gibberellins, cytokinin brassinolide (An and Mou, 2011). Most phytohormones have been reported to trigger PI3K signaling. In seed germination, PI3K is a positive regulator of GA signaling (Liu et al., 2012). And ABA and Auxin-induced ROS production also requires the activation of PI3K (Park et al., 2003; Joo et al., 2005). Moreover, PI3K is involved in MeJA-induced leaf senescence (Hung et al., 2006; Hung and Kao, 2007; Liu et al., 2016). Increasing evidences indicated that PI3K functioned as a common platform for multi-hormone signaling to trigger intracellular response (Hirsch et al., 2007). For network regulation of phytohormone in plant immunity, PI3K seems to play a complex role in plant-pathogen interactions. We speculated that the timing of infection might be a crucial element in the regulatory role of PI3K on pathogen defense.

The role of ABA in plant immunity is complex. In *Arabidopsis*, ABA-regulated stomatal closure is a key element of pre-invasion SA-regulated innate immunity to *Pseudomonas syringae* (Melotto et al., 2006). COR counteracts PAMP-induced stomatal closure downstream of ABA (Xie et al., 1998). And our previous study showed that one unknown upstream signaling of PI3K initiated the antagonistic effect on JA signaling (Liu et al., 2016). Although gene expression of *AtPI3K* was inhibited by ABA upon syringe-infiltrated with *Pst* DC3000 (*avrRpt2*) (**Figure 1**), we cannot rule out the possibility that ABA regulated the PI3K signaling in stomatal immunity. Moreover, guard cell ABA could activate ROS-generating NADPH oxidases (Kwak et al., 2006), which are also necessary for ROS production during pathogen defense (Torres and Dangl, 2005). However, the exact role of ABA on PI3K signaling should be determined by future.

Stomata functions as innate immunity gates to actively prevent bacterial entry in plant immunity (Melotto et al., 2006). If *Pst* DC3000 fails to enter leaf tissues after spray-inoculation, it dies quickly (Xin and He, 2013). In eukaryocyte, the activation of PI3K signaling is tightly associated with primary metabolism. Upon glucose stimulation, PI3K regulated V-ATPase activation (Sautin et al., 2005). PI(3)P production by VPS34 is stimulated by amino acid (Byfield et al., 2005; Yoon et al., 2016). And previous study also revealed a significant change of primary metabolites in bacterial invasion. Fructose showed more than a threefold increase at 30 min, and most amino acids showed a decrease at 180 min after pathogen infection (Pang et al., 2018). Therefore, we speculated that PI3K signaling might be activated by sugar and amino acid signaling in the early stage of bacterial invasion. Once the accumulation of sugar and amino acid was decreased, PI3K signaling was diminished. Thus, we found the overexpression of PI3K recovered to almost the same level as control plants in stomatal reopening. However, the enhanced closure of stomata induced by PI3K in the early stage of bacterial invasion is still very important for plant defense. For one thing, PI3K signaling senses the danger of potential bacterial invasion more effectively and take a more effective restriction of bacterial entry through the epidermis. For another, this stomatal regulation might be necessary for the priming induction (Ramirez-Prado et al., 2018). Primed plants are in a heightened state of defense and produce a stronger defensive response when challenged (Singh et al., 2012).

Our results revealed ROS content was enhanced by overexpression of *AtVPS34* after syringe infiltration or spray inoculation with *Pst* DC3000 (*avrRpt2*). ROS production is thought to be directly toxic to pathogens in animal immunity (Lambeth, 2004), and restricts pathogen entry by triggering stomatal closure (Su et al., 2017). It seems that PI3K regulates stomatal immunity by promoting ROS accumulation. There is multiple pathways for ROS production in guard cells (Song et al., 2014). Our former study revealed that PI3K could regulate the activity of NADPH oxidase in seed germination (Liu et al., 2012). And upon perception of PAMPs, NADPH oxidase RbohD could be activated by plasma-associated kinase BIK1 to induce ROS production (Su et al., 2017). We speculated that NADPH oxidase was one of the important sources of ROS regulated by PI3K. ROS burst triggers an activation of MAPKs signaling and an increase in the concentration of cytosolic calcium, leading to activation of ion channels and modification of cellular turgor, thus closure of the stomatal pores (Balmant et al., 2016). Moreover, cytoskeleton plays a vital role in *Arabidopsis* guard cell architecture, thus pathogen manipulation of actin within the stomata might be implicated in having a role in immune subversion (Porter and Day, 2015). Growing evidence showed that PI3K and its production PI3P modulate actin filament reorganization (Choi et al., 2008; Li et al., 2008). Thus, PI3K might regulate stomatal immunity by modulating the dynamic of cytoskeleton. Of course, all these speculations should be investigated in future studies.

Once bacteria enter leaf tissues, SA possibly enhances PI3K signaling. Our experiment showed that *AtVPS34* was expressed in leaf epidermal and guard cells, especially in guard cell. And exogenous SA further enhanced PI3K expression in stomata after syringe infiltration. It seems that PI3K-mediated guard cell signaling regulates not only stomatal immunity, but also other type of plant defense. Previous study showed ETI is an accelerated and amplified PTI response, resulting in disease resistance and usually an HR PCD at the infection site (Jones and Dangl, 2006). Plant autophagy operated negative feedback loop modulating SA signaling to negatively regulate immunity-related PCD (Yoshimoto et al., 2009). Therefore, SA is hyperaccumulated in L-methionine sulfoximine-induced cell death in *NbPI3K*-RNAi plant (Sumida et al., 2017). However, *AvrRpt2*-triggered HR PCD may be independent of autophagy and may require other cell death processes (Hofius et al., 2009). Thus, the negative feedback of PI3K on the SA production might not be existed in avirulent *Pst* DC3000 (*AvrRpt2*) infected *Arabidopsis*. Nevertheless, the effect of PI3K on the ROS production is important. Therefore, enhancement the ROS content and expression of genes encoding antioxidant enzyme by overexpression of *AtVPS34* in the process of *Pst* DC3000 (*avrRpt2*) infection (*avrRpt2*) (**Figure 4**) might result in initiation of *PR1* and *PR5* gene expression. Plant immunity to avirulent pathogen is usually associated with subcellular membrane dynamics, such as fusion between the vacuolar and plasma membranes (Hatsugai et al., 2018). And previous study has been revealed that YFP-2xFYVE, a fluorescent PI3P-specific biosensor, strongly labelled the vacuolar membrane in leaf epidermal and guard cells (Vermeer et al., 2006). Therefore, it is interesting to investigate the role of PI3K on membrane traffic in plant resistance to avirulent bacteria invasion in the future.

In this study, previously unknown role of PI3K in bacterial resistance was unraveled. Our results showed that PI3K promotes the process of stomatal immunity and play a positive role in SA-induced immunity possibly *via* the regulation of ROS production. We found that PI3K could play a positive role in *Arabidopsis* against avirulent *Pst* DC3000 (*avrRpt2*) and *Pst* DC3000 infection. Given a key role of PI3K in plant immunity, we propose that analysis of the effect of PI3K on cytoskeleton dynamic and ROS production may provide novel important information for the control of bacterial disease.

## Data Availability Statement

All datasets generated for this study are included in the article/**Supplementary Material**.

## Author Contributions

HZ and JL designed the research. HZ, XL, XZ, NQ, KX, WY, YZ and JL performed research. HZ, YS, RZ and JL contributed analytic and computational tools. HZ, XL, XZ, NQ, KX, WY, YZ, YS, RZ and JL analyzed data. HZ, RZ and JL wrote the paper. All authors read and approved the final manuscript.

## Funding

This research was supported by the China Postdoctoral Science Foundation (2019M652233), the Natural Science Foundation of Fujian Province of China (2016J01102; 2017J01425), the Young and Middle-aged Teachers Education Scientific Research Project of Fujian Provincial Department of Education (JAT160163; JAT160176), the Fujian Agriculture and Forestry University (Xjq201617), and the School International Science and Technology Cooperation and Exchange Project (KXB16009A).

## Conflict of Interest

The authors declare that the research was conducted in the absence of any commercial or financial relationships that could be construed as a potential conflict of interest.
