# Supplementary material for: Phosphoinositide 3-Kinase Promotes Oxidative Burst, Stomatal Closure and Plant Immunity in Bacterial Invasion
Source: Front Plant Sci. 2020 Jan 24;10:1740. doi: 10.3389/fpls.2019.01740 (PMC7025545; doi:10.3389/fpls.2019.01740)
Supplement: Supplementary file 1 [file DataSheet_1.doc]

Supplementary data


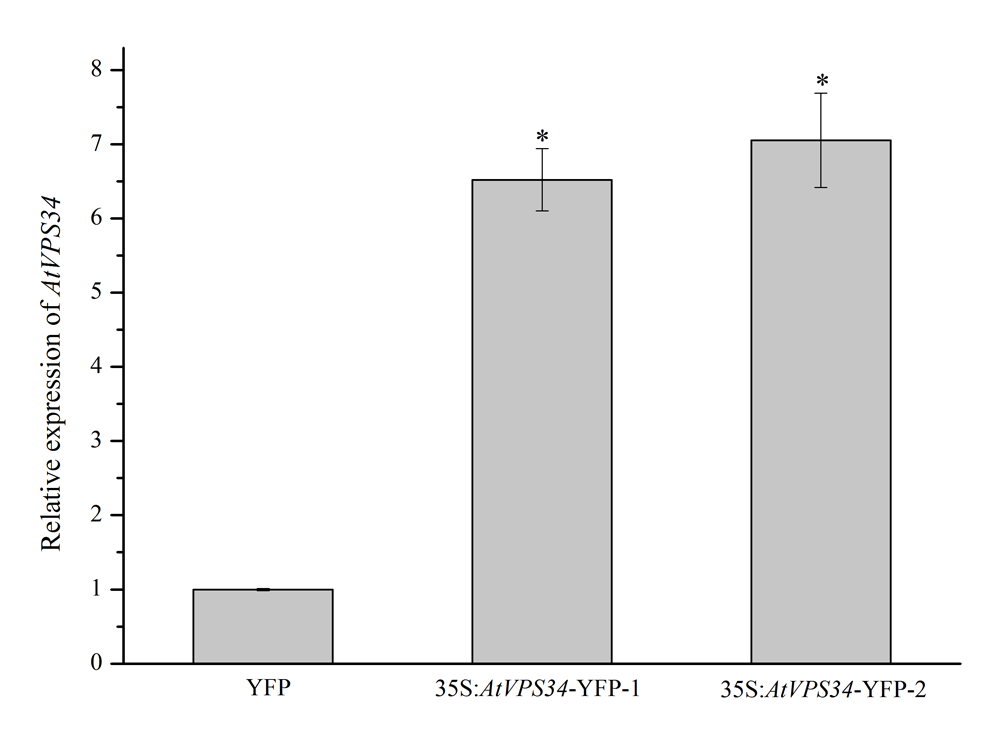


**Figure S1. qRT-PCR determination of transgenic *Arabidopsis*.** Total RNA was extracted from three-week-old seedlings of 35Spro::YFP and 35Spro::*AtVP34*-YFP lines, Arabidopsis followed by RT-PCR analysis. *AtActin2* was used as an internal control. Asterisks (*) indicate significant differences from 35Spro::YFP *Arabidopsis* at P<0.05. Each bar is the mean ± SD of three biological replications.


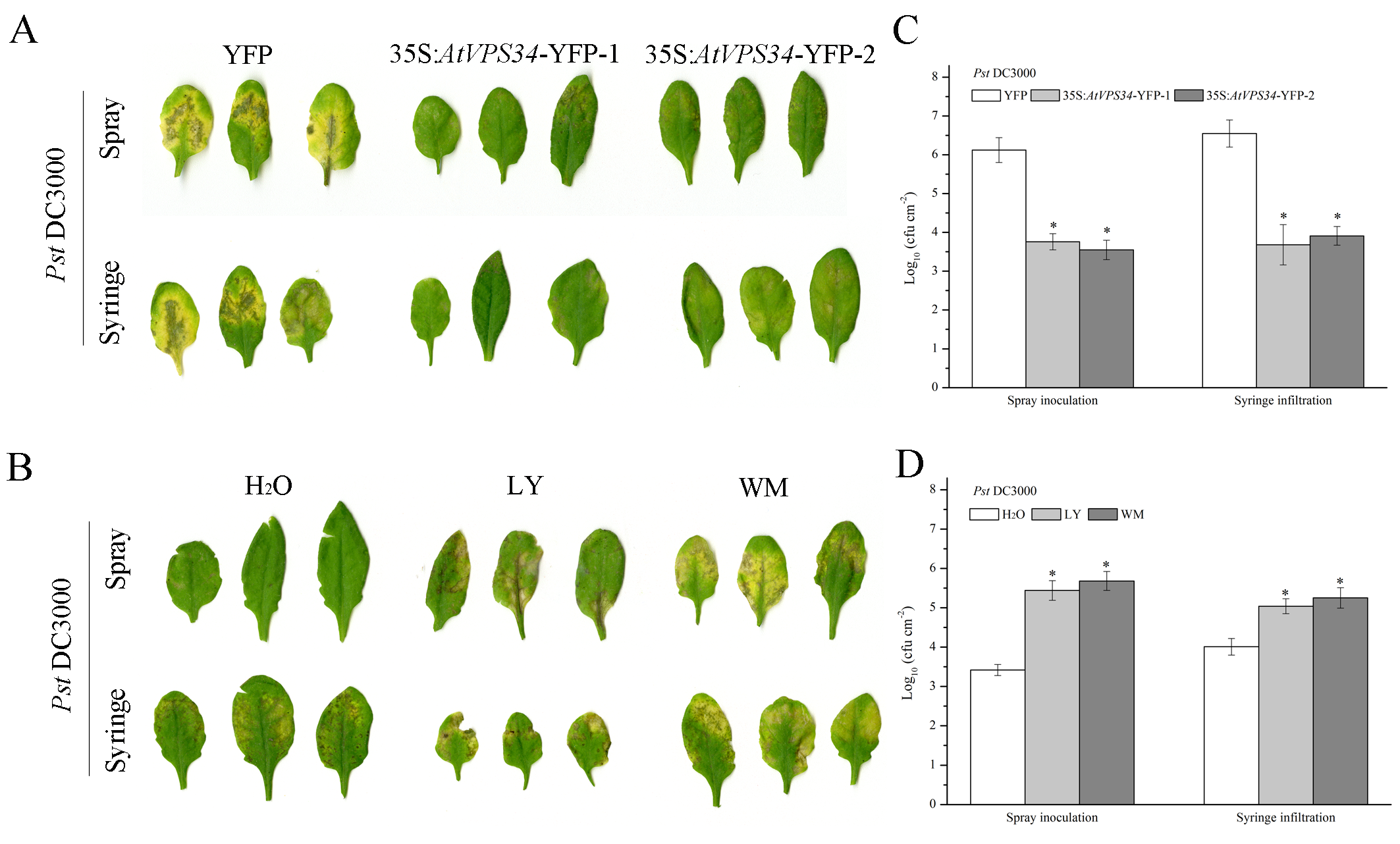


**Figure S2. PI3K functioned in plant immunity against *Pst* DC3000.** (A) 35Spro::*AtVP34*-YFP *Arabidopsis* and 35Spro::YFP *Arabidopsis* were spray-inoculated or syringe-infiltrated with *Pst* DC3000 for 2 d, Lesion phenotypes in *Arabidopsis* leaves was taken. Pictures represent typical examples. And the corresponding bacterial growth quantification of spray-inoculated or syringe-infiltrated leaves was shown in (C). Each bar is the mean ± SD of three biological replications. Asterisks (*) indicates significant difference by t test from 35Spro::YFP *Arabidopsis* (P<0.05; Student’s t test). (B) WT *Arabidopsis* was pretreated either with 30 μM LY or 10 μM WM or not for 1 d, then syringe-infiltrated or spray-inoculated with *Pst* DC3000 for 1 d. Lesion phenotypes in *Arabidopsis* leaves was taken. Pictures represent typical examples. And the corresponding bacterial growth quantification of spray-inoculated or syringe-infiltrated leaves was shown in (D). Each bar is the mean ± SD of three biological replications. Asterisks (*) indicates significant difference by t test from no PI3K inhibitors treatment (P<0.05; Student’s t test).


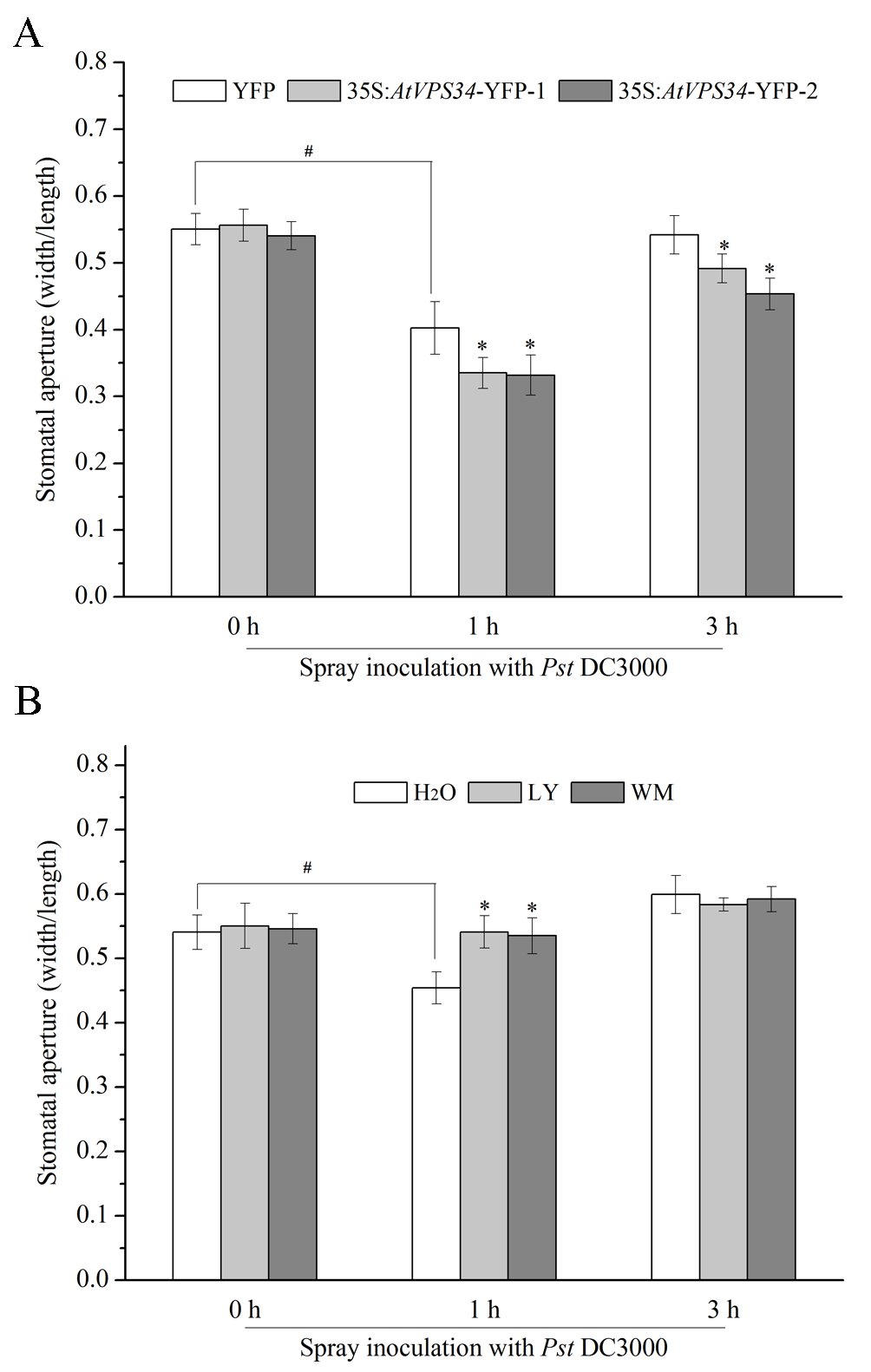


**Figure S3. PI3K functioned in stomatal immunity against *Pst* DC3000.** (A) 35Spro::*AtVP34*-YFP *Arabidopsis* and 35Spro::YFP *Arabidopsis* were taken in light for at least 3 h, (B) WT *Arabidopsis* was pretreated either with 30 μM LY or 10 μM WM or not for 24 h, then transport the plant under light for at least 3 h. The full expanded young leaves were immersed in water or *Pst* DC3000 suspension (10^8^ cfu/ml in water). 1 and 3 h after spray inoculation, epidermis of three leaves was peeled off and immediately observed under a microscope. The stomatal aperture was represented as the ratio of width to length. Each bar is the mean ± SD of three biological replications (n˃30). Asterisks (*) indicate statistically significant differences from control in the indicated times (P<0.05; Student’s t test). Hash marks (#) indicate statistically significant differences between indicated samples (P<0.05; Student’s t test).
